# Genome-wide survey of the dehydrin genes in bread wheat (*Triticum aestivum* L.) and its relatives: identification, evolution and expression profiling under various abiotic stresses

**DOI:** 10.1186/s12864-022-08317-x

**Published:** 2022-01-23

**Authors:** Yongchao Hao, Ming Hao, Yingjie Cui, Lingrang Kong, Hongwei Wang

**Affiliations:** 1 State Key Laboratory of Crop Biology, College of Agronomy, Shandong Agricultural University, Taian, 271018 China; 2 College of Forestry, Shandong Agricultural University, Taian, 271018 China

**Keywords:** Bread wheat, *DHN* gene family, Expression profile, Biotic stress, Abiotic stress

## Abstract

**Background:**

Bread wheat (*Triticum aestivum*) is an important staple cereal grain worldwide. The ever-increasing environmental stress makes it very important to mine stress-resistant genes for wheat breeding programs. Therefore, *dehydrin* (*DHN*) genes can be considered primary candidates for such programs, since they respond to multiple stressors.

**Results:**

In this study, we performed a genome-wide analysis of the *DHN* gene family in the genomes of wheat and its three relatives. We found 55 *DHN* genes in *T. aestivum*, 31 in *T. dicoccoides*, 15 in *T. urartu*, and 16 in *Aegilops tauschii*. The phylogenetic, synteny, and sequence analyses showed we can divide the *DHN* genes into five groups. Genes in the same group shared similar conserved motifs and potential function. The tandem *TaDHN* genes responded strongly to drought, cold, and high salinity stresses, while the non-tandem genes respond poorly to all stress conditions. According to the interaction network analysis, the cooperation of multiple DHN proteins was vital for plants in combating abiotic stress.

**Conclusions:**

Conserved, duplicated *DHN* genes may be important for wheat being adaptable to a different stress conditions, thus contributing to its worldwide distribution as a staple food. This study not only highlights the role of *DHN* genes help the *Triticeae* species against abiotic stresses, but also provides vital information for the future functional studies in these crops.

**Supplementary Information:**

The online version contains supplementary material available at 10.1186/s12864-022-08317-x.

## Background

Bread wheat (*Triticum aestivum*) is an important staple cereal crop providing ~ 20% of the global dietary protein and calories [1, 2]. It comprises three homologous sub-genomes (AABBDD; 2n = 6x = 42) originating from two natural hybridization events [3, 4]. First, tetraploidization from the hybridization between *T. urartu* (AA; 2n = 2x = 14) and an unknown close relative of *Aegilops speltoides* (BB; 2n = 2x = 14) generated the tetraploid wild emmer wheat (*T. turgidum* ssp. *dicoccoides*, AABB, 2n = 4x = 28). Wild emmer wheat hybridized with *Ae. tauschii* (DD; 2n = 2x = 14) about 8000 years ago to produce hexaploid bread wheat [5]. Environmental stressors including abiotic stressors (e.g., drought, salinity, and high and low temperatures) [6–8] and biotic stressors like *Fusarium graminearum* (*Fusarium* head blight or FHB), *Blumeria graminis* (powdery mildew), and *Puccinia striiformis* (stripe rust) challenge bread wheat yield during its growth phase [9]. The key to facing these challenges is mining for stress-resistant genes and utilizing them for breeding. With the release of the genome assembly and annotation for *T. aestivum* [10], *T. dicoccoides* [11], *T. urartu* [12], and *Ae. tauschii* [13], a genome-wide analysis of all stress-related genes in wheat and its relatives can now be realized. Furthermore, large-scale RNA sequencing (RNA-seq) provides a rich resource for analyzing their related gene expression patterns not only under diverse stress conditions but also at different developmental stages [14].

Dehydrins (DHNs) are a class of highly hydrophilic, stress-responsive proteins rich in charged and polar amino acids [15, 16]. These proteins accumulate during late embryogenesis and are induced in vegetative tissues by several cell-dehydrating environmental stressors like drought, salinity, and cold [17]. Based on their sequence characteristics, DHNs are defined as proteins containing at least one copy of a conserved motif called the K-segment [18, 19]. The K-segment (consensus EKKGIM [E/D]KIKEKLPG) is a lysine-rich amino acid sequence, forming amphiphilic α-helixes at the protein’s C-terminus [20, 21]. DHNs also possess other conserved motifs, like the N-terminal tyrosine-rich Y-segment (consensus [T/V] D [E/Q]YGNP), and the serine-rich S-segment (consensus LHRSGS4–10(E/D)3) containing a stretch of 4–10 serine residues [22, 23]. The diversity of the conserved domains allows the DHN gene to form combinations of different domains, and then produce different groups [24, 25]. Based on the presence of these conserved motifs (K-, S-, and Y-segment), DHNs are classified into different categories of YnSKn, YnKn, SKn, KnS, and Kn [18, 19, 26].

DHNs are stress proteins protecting plants against dehydration by: (a) binding metal ions and scavenging reactive oxygen species, (b) binding DNA or phospholipids to maintain biological activity, (c) binding proteins to prevent denaturation, and (d) holding water molecules [27, 28]. DHN family members are intrinsically unstructured, heat-stable proteins expressed during the late embryogenesis stage [29, 30]. Their characteristic protein conformational changes result in protein functional changes via a phenomenon called ‘moonlighting’, and thus also called IDPs/IUPs (intrinsically unstructured/disordered proteins). They either may help in forming and stabilizing the plant cytoplasmic glassy state during dehydration, or serve as hub proteins coordinating cellular signaling crosstalk involved in the stress response [31, 32]. Previous studies demonstrated that DHNs are crucial in abiotic stress tolerance; overexpressing the *Solanum habrochaites DHN* gene enhanced transgenic tomato tolerance against multiple abiotic stressors; overexpressing the oleaster *DHN* gene *OesDHN* improved drought tolerance in *Arabidopsis*; overexpression of four *Prunus mume DHN*s in *Escherichia coli* and tobacco resulted in increased freezing resistance; *HbDHN1* and *HbDHN2* from *Hevea brasiliensis* significantly increased drought, salt, and osmotic stress tolerance when overexpressed in *Arabidopsis* [33–36]. These studies indicate the extensive involvement of plant DHNs in abiotic stress tolerance. Several studies have shown that DHNs might also play important roles in both plant development and biotic stress response. For example, *Medicago truncatula* Y2K4-type dehydrin (MtCAS31) interacts with AtICE1, which is essential for stomatal development [37]; expression of several *DHN*s in drought-tolerant oak species *Quercus ilex* are induced by a *Phytophthora cinnamomic* infection [38].

In this study, we identified the *DHN* genes and its homologs in bread wheat and its relatives and analyzed their phylogenetic, syntenic, and sequence relationships. We analyzed the putative promoter *cis*-elements of the *TaDHN* genes. Then, we investigated the expression profiles of the *DHN* gene family in response to various environmental stressors (including biotic and abiotic stressors) and hormones. Finally, we analyzed the interaction network of *DHN* genes and experimentally verified their predicted subcellular location. Therefore, this study (a) provides a comprehensive structural and functional analysis of the *DHN* gene family in bread wheat and its relatives, and (b) clarifies the important role of *DHN* genes help against various abiotic stresses.

## Results

### Characterization of DHN genes in bread wheat and its relatives

We used HMMER 3.1 and BLASTP for searching *DHN* genes in the genomes of bread wheat and its relatives, based on the Pfam database-derived HMM profile of the DHN domain (PF00257) as a query. Then, we verified the predicted sequences using InterPro and CDD. Finally, we identified 117 putative *DHN* genes. Among them, we detected 55 *DHN* genes in *T. aestivum*, 31 in *T. dicoccoides*, 15 in *T. urartu*, and 16 in *Ae. tauschii*. These *DHN* gene numbers are directly related to the genome ploidy. The *DHN* gene names, locus IDs, and other features are shown in Table 1.

**Table 1:** The details of DHN genes among bread wheat and its relatives

Gene Name	Locus ID	Type	Genomic Position	BP	GC (%)	AA	MW (kDa)	pI	Subcellular Localization
*TaDHN1-A*	*TraesCS3A02G254600*	YSK2	476,563,869–476,564,968(−)	642	68.07	213	21.83	6.75	Cytoplasm Nucleus
*TaDHN1-B*	*TraesCS3B02G286600*	YSK2	458,398,889–458,399,630(−)	654	67.13	217	22.3	7.5	Cytoplasm Nucleus
*TaDHN1-D*	*TraesCS3D02G255500*	YSK2	357,146,923–357,147,959(−)	648	67.75	215	22.24	7.19	Cytoplasm Nucleus
*TaDHN2-A*	*TraesCS3A02G396200*	YSK3	643,459,970–643,461,316(−)	828	67.63	275	27.02	10.13	Cytoplasm
*TaDHN2-B*	*TraesCS3B02G428200*	YSK3	667,112,076–667,113,352(−)	825	68.12	274	27.19	10.29	Cytoplasm
*TaDHN2-D*	*TraesCS3D02G390200*	YSK3	505,318,572–505,319,988(−)	828	67.87	275	27.16	10.26	Cytoplasm
*TaDHN3-A*	*TraesCS4A02G250900*	Y2SK3	562,289,788–562,291,566(−)	1368	70.37	455	43.74	9.28	Cytoplasm
*TaDHN3-B*	*TraesCS4B02G064200*	Y2SK3	57,136,426–57,138,194(−)	1374	69.92	457	43.89	9.27	Cytoplasm
*TaDHN3-D*	*TraesCS4D02G063100*	Y2SK3	39,233,033–39,234,840(−)	1293	70.15	430	41.22	9.47	Cytoplasm
*TaDHN4-A1*	*TraesCS5A02G369800*	YSK2	569,677,389–569,678,193(+)	432	71.53	143	14.57	8.91	Cytoplasm
*TaDHN4-A2*	*TraesCS5A02G369900*	YSK2	569,682,833–569,683,707(+)	423	70.92	140	14.24	8.91	Cytoplasm
*TaDHN4-B1*	*TraesCS5B02G372100*	YSK2	550,320,429–550,321,418(+)	432	71.76	143	14.43	8.91	Cytoplasm
*TaDHN4-B2*	*TraesCS5B02G372200*	YSK2	550,337,855–550,338,611(+)	417	70.5	138	14.22	8.91	Cytoplasm
*TaDHN4-D1*	*TraesCS5D02G379200*	YSK2	450,373,636–450,374,483(+)	432	71.53	143	14.52	8	Cytoplasm
*TaDHN4-D2*	*TraesCS5D02G379300*	YSK2	450,379,533–450,380,460(+)	402	69.65	133	13.93	9.44	Cytoplasm
*TaDHN5-A1* ^*a*^	*TraesCS5A02G424700*	YSK1	610,078,219–610,079,136(−)	336	67.26	111	11.48	10.15	Cytoplasm
*TaDHN5-A2*	*TraesCS5A02G424800*	YSK2	610,184,778–610,185,696(−)	450	65.56	149	15.22	9.96	Cytoplasm
*TaDHN5-B1*	*TraesCS5B02G426700*	YSK2	602,483,279–602,484,206(−)	453	66	150	15.18	10.16	Cytoplasm
*TaDHN5-B2*	*TraesCS5B02G426800*	YSK2	602,648,556–602,649,390(−)	453	65.78	150	15.22	9.99	Cytoplasm
*TaDHN5-D1*	*TraesCS5D02G433200*	YSK2	489,012,960–489,013,838(−)	459	66.67	152	15.35	10.16	Cytoplasm
*TaDHN5-D2*	*TraesCS5D02G433300*	YSK2	489,166,583–489,167,421(−)	465	66.67	154	15.59	10.16	Cytoplasm
*TaDHN6-A*	*TraesCS6A02G059800*	YSK2	31,583,535–31,584,464(−)	462	68.4	153	15.51	9.46	Cytoplasm
*TaDHN6-B*	*TraesCSU02G086200*	YSK2	76,960,851–76,961,538(+)	456	67.76	151	15.29	9.68	Cytoplasm
*TaDHN6-D* ^*a*^	*TraesCSU02G122200*	YSK1	104,041,218–104,042,344(−)	450	69.56	149	14.86	7.5	Cytoplasm
*TaDHN7-A*	*TraesCS6A02G253300*	SK3	468,473,627–468,475,112(−)	807	64.19	268	28.82	5.05	Nucleus
*TaDHN7-B*	*TraesCS6B02G273400*	SK3	493,352,704–493,354,073(−)	780	62.69	259	27.97	4.98	Nucleus
*TaDHN7-D*	*TraesCS6D02G234700*	SK3	329,080,938–329,082,415(−)	789	63.88	262	28.16	4.97	Nucleus
*TaDHN8-A* ^*a*^	*TraesCS6A02G350100*	K3	581,982,926–581,983,580(+)	573	65.1	190	19.24	7.74	Cytoplasm
*TaDHN8-B*	*TraesCS6B02G383200*	K6	658,177,094–658,178,907(+)	1218	66.56	405	40.29	7.37	Cell wall Cytoplasm Nucleus
*TaDHN8-D* ^*a*^	*TraesCS6D02G332500*	K3	434,811,674–434,812,738(+)	540	66.3	179	17.8	8.23	Cytoplasm
*TaDHN9-A*	*TraesCS6A02G350200*	K2	582,086,438–582,087,160(+)	282	62.06	93	9.66	7.43	Cytoplasm
*TaDHN9-D*	*TraesCS6D02G332600*	K2	435,012,400–435,012,681(+)	282	63.12	93	9.66	7.43	Cytoplasm
*TaDHN10-A*	*TraesCS6A02G350300*	K14	582,092,081–582,096,138(+)	2976	62.96	991	101.61	6.33	Cytoplasm
*TaDHN10-B* ^*a*^	*TraesCS6B02G695200LC*	K8	658,234,035–658,241,687(+)	1671	61.88	556	57.44	6.67	Cytoplasm
*TaDHN10-D*	*TraesCS6D02G332700*	K12	435,072,895–435,075,633(+)	2739	63.08	912	93.49	6.45	Cell wall Cytoplasm Nucleus
*TaDHN11-A*	*TraesCS6A02G350500*	YSK2	582,264,726–582,266,027(+)	666	68.92	221	22.05	9.45	Cell wall Cytoplasm Nucleus
*TaDHN11-B*	*TraesCS6B02G383500*	YSK2	658,402,976–658,404,180(+)	690	69.13	229	23.01	9.79	Cell wall Cytoplasm Nucleus
*TaDHN11-D*	*TraesCS6D02G332900*	YSK2	435,351,033–435,352,218(+)	651	69.12	216	21.62	9.5	Cell wall Cytoplasm Nucleus
*TaDHN12-A1*	*TraesCS6A02G350600*	YSK2	582,511,276–582,512,221(+)	489	66.87	162	16.28	9.88	Cytoplasm
*TaDHN12-A2*	*TraesCS6A02G350700*	YSK2	582,516,436–582,517,359(+)	459	64.49	152	15.52	8.05	Cytoplasm
*TaDHN12-A3*	*TraesCS6A02G350800*	SK2	582,630,751–582,631,295(−)	432	63.66	143	14.82	9.88	Cytoplasm
*TaDHN12-A4* ^*b*^	*TraesCS6A02G350900*	YSK1	582,638,141–582,638,862(−)	573	64.92	190	20.14	11.34	Cytoplasm
*TaDHN12-B1*	*TraesCS6B02G695700LC*	YSK2	658,477,430–658,478,020(+)	489	63.27	162	16.13	9.79	Cytoplasm
*TaDHN12-B2*	*TraesCS6B02G695800LC*	YSK2	658,496,215–658,496,805(+)	489	63.27	162	16.13	9.79	Cytoplasm
*TaDHN12-B3*	*TraesCS6B02G695900LC*	YSK2	658,515,366–658,515,956(+)	489	63.27	162	16.1	9.79	Cytoplasm
*TaDHN12-B4*	*TraesCS6B02G383600*	YSK2	658,530,539–658,531,483(+)	477	66.46	158	15.84	9.69	Cytoplasm
*TaDHN12-B5*	*TraesCS6B02G383800*	YSK2	658,577,562–658,578,499(−)	501	65.67	166	16.7	8.89	Cytoplasm
*TaDHN12-D1*	*TraesCS6D02G333000*	YSK2	435,711,668–435,712,623(+)	489	66.87	162	16.2	8.93	Cytoplasm
*TaDHN12-D2*	*TraesCS6D02G333100*	YSK2	435,749,803–435,750,719(+)	435	65.06	144	14.51	9.79	Cytoplasm
*TaDHN12-D3*	*TraesCS6D02G333200*	YSK2	435,763,700–435,764,598(+)	468	65.6	155	15.73	9.41	Cytoplasm
*TaDHN12-D4*	*TraesCS6D02G333300*	YSK2	435,831,506–435,832,386(−)	483	66.87	160	16.26	8.94	Cytoplasm
*TaDHN12-D5*	*TraesCS6D02G333600*	YSK2	435,962,275–435,963,310(−)	504	66.27	167	16.71	8.05	Cytoplasm
*TaDHN13-A*	*TraesCS7A02G560000*	K3	731,882,428–731,883,023(+)	375	61.87	124	12.83	8.08	Cytoplasm
*TaDHN13-B*	*TraesCS7B02G484900*	K3	741,668,510–741,668,887(+)	378	64.02	125	12.67	7.04	Cytoplasm
*TaDHN13-D*	*TraesCS7D02G549900*	K2	634,439,606–634,440,272(−)	339	63.13	112	11.53	6.8	Cytoplasm
*TdDHN1-A*	*TRIDC3AG038190*	YSK2	483,446,623–483,451,982(−)	660	67.42	219	22.53	6.93	Cytoplasm Nucleus
*TdDHN1-B*	*TRIDC3BG042940*	YSK2	468,417,850–468,427,038(−)	654	66.97	217	22.37	7.19	Cytoplasm Nucleus
*TdDHN2-A* ^*a*^	*TRIDC3AG056410*	YSK2	639,664,417–639,665,447(−)	390	64.36	129	13.24	10.56	Cytoplasm Nucleus
*TdDHN2-B* ^*a*^	*TRIDC3BG063150*	YSK2	677,652,490–677,653,501(−)	405	65.43	134	13.87	10.69	Cytoplasm
*TdDHN3-A* ^*a*^	*TRIDC4AG039320*	SK3	555,522,541–555,523,348(−)	552	66.67	183	18.37	10.19	Cytoplasm
*TdDHN3-B* ^*a*^	*TRIDC4BG009930*	Y2SK3	55,083,350–55,084,815(−)	543	65.38	180	18.02	10.19	Cytoplasm
*TdDHN4-A1*	*TRIDC5AG054100*	YSK2	564,921,473–564,922,000(+)	432	71.76	143	14.56	8.91	Cytoplasm
*TdDHN4-A2*	*TRIDC5AG054110*	YSK2	564,926,944–564,927,492(+)	423	70.92	140	14.27	8.87	Cytoplasm
*TdDHN4-B1*	*TRIDC5BG058060*	YSK2	556,085,190–556,085,719(+)	432	71.76	143	14.43	8.91	Cytoplasm
*TdDHN4-B2*	*TRIDC5BG058080*	YSK2	556,101,420–556,101,927(+)	417	70.26	138	14.25	8.91	Cytoplasm
*TdDHN5-A1* ^*a*^	*TRIDC5AG061380*	SK1	605,402,094–605,402,742(−)	219	63.01	72	7.61	10.81	Nucleus
*TdDHN5-A2* ^*b*^	*TRIDC5AG061420*	YSK2	605,513,222–605,513,988(−)	522	63.03	173	17.79	10.35	Cytoplasm
*TdDHN5-B*	*TRIDC5BG065560*	YSK2	608,742,508–608,743,380(−)	456	65.79	151	15.37	10.13	Cytoplasm
*TdDHN6-A*	*TRIDC6AG007480*	YSK2	31,000,232–31,000,891(−)	462	67.97	153	15.44	8.93	Cytoplasm
*TdDHN6-B*	*TRIDC6BG010780*	YSK2	55,591,718–55,592,361(−)	456	67.76	151	15.29	9.68	Cytoplasm
*TdDHN7-A*	*TRIDC6AG039020*	SK3	470,140,072–470,141,622(−)	807	64.19	268	28.82	5.05	Nucleus
*TdDHN7-B*	*TRIDC6BG045690*	SK3	486,136,098–486,141,800(+)	780	62.56	259	27.94	4.98	Nucleus
*TdDHN8-A* ^*a*^	*TRIDC6AG052540*	K4	582,467,279–582,468,478(+)	792	68.06	263	26.21	7.44	Cytoplasm
*TdDHN8-B* ^*b*^	*TRIDC6BG061300*	K7	642,747,123–642,748,606(+)	1410	66.67	470	47.07	7.63	Cell wall Cytoplasm Nucleus
*TdDHN9-A*	*TRIDC6AG052550*	K2	582,534,354–582,534,931(+)	282	62.06	93	9.66	7.43	Cytoplasm
*TdDHN9-B* ^*b*^	*TRIDC6BG061310*	K2	642,748,977–642,749,569(+)	354	65.82	117	11.67	7.54	Cytoplasm
*TdDHN10-A* ^*a*^	*TRIDC6AG052570*	K8	582,541,438–582,543,392(+)	1698	61.37	565	58.87	6.45	Cytoplasm
*TdDHN11-A* ^*a*^	*TRIDC6AG052590*	YSK2	582,698,877–582,699,685(+)	489	67.28	162	16.66	9.6	Cell wall Cytoplasm Nucleus
*TdDHN11-B*	*TRIDC6BG061340*	YSK2	643,041,875–643,042,827(+)	696	69.25	231	23.13	9.45	Cell wall Cytoplasm Nucleus
*TdDHN12-A1*	*TRIDC6AG052630*	YSK2	582,893,775–582,898,592(+)	489	66.87	162	16.22	9.13	Cytoplasm
*TdDHN12-A2*	*TRIDC6AG052640*	YSK2	582,992,994–582,993,790(−)	474	64.14	157	16.14	8.08	Cytoplasm
*TdDHN12-A3*	*TRIDC6AG052650*	YSK2	583,001,187–583,001,807(−)	441	65.76	146	14.75	9.79	Cytoplasm
*TdDHN12-B1*	*TRIDC6BG061350*	YSK2	643,116,359–643,132,143(+)	477	66.67	158	15.87	9.71	Cytoplasm
*TdDHN12-B2*	*TRIDC6BG061380*	YSK2	643,190,550–643,191,397(−)	501	65.87	166	16.65	8.89	Cytoplasm
*TdDHN13-A*	*TRIDC7AG077740*	K3	723,743,247–723,743,983(−)	375	61.87	124	12.83	8.08	Cytoplasm
*TdDHN13-B*	*TRIDC7BG076250*	K3	750,614,280–750,614,913(+)	381	63.78	126	12.8	7.38	Cytoplasm
*TuDHN1*	*TuG1812G0300003021.01*	YSK2	477,045,519–477,046,650(−)	660	67.42	219	22.57	7.14	Cytoplasm Nucleus
*TuDHN3*	*TuG1812G0400000583.01*	Y2SK3	41,901,253–41,903,075(−)	1392	70.55	463	44.55	9.28	Cytoplasm
*TuDHN4–1*	*TuG1812G0500003981.01*	YSK2	535,938,718–535,939,515(+)	432	71.53	143	14.55	8.03	Cytoplasm
*TuDHN4–2*	*TuG1812G0500003982.01*	YSK2	535,944,152–535,945,001(+)	423	71.16	140	14.24	8.91	Cytoplasm
*TuDHN5–1* ^*a*^	*TuG1812G0500004492.01*	YSK1	574,218,374–574,219,129(−)	336	67.26	111	11.46	9.8	Cytoplasm
*TuDHN5–2* ^*a*^	*TuG1812S0001634800.01*	YSK1	1–593(−)	414	67.87	138	13.91	7.97	Cytoplasm
*TuDHN6*	*TuG1812G0600000621.01*	YSK2	31,890,359–31,891,122(−)	462	68.18	153	15.4	8.1	Cytoplasm
*TuDHN7*	*TuG1812G0600002817.01*	SK3	439,323,348–439,324,718(−)	807	64.06	268	28.85	5.05	Nucleus
*TuDHN8*	*TuG1812G0600003768.01*	K6	539,540,401–539,541,995(+)	1176	68.54	391	38.82	7.48	Cell wall Cytoplasm Nucleus
*TuDHN9*	*TuG1812G0600003769.01*	K2	539,598,064–539,599,229(+)	282	62.06	93	9.66	7.43	Cytoplasm
*TuDHN11*	*TuG1812G0600003775.01*	YSK2	539,946,480–539,947,767(+)	756	68.65	251	24.98	9.12	Cell wall Cytoplasm Nucleus
*TuDHN12–1* ^*b*^	*TuG1812S0003423700.01*	YSK1	1979–2944(+)	537	67.6	178	17.93	10.89	Cytoplasm
*TuDHN12–2*	*TuG1812S0003424100.01*	YSK2	5679–6605(+)	459	64.27	152	15.64	8.89	Cytoplasm
*TuDHN12–3*	*TuG1812G0600003779.01*	YSK2	540,160,098–540,161,020(−)	510	67.06	169	16.94	8.89	Cytoplasm
*TuDHN13*	*TuG1812G0700005988.01*	K3	712,117,702–712,118,431(+)	375	61.87	124	12.74	7.47	Cytoplasm
*AetDHN1*	*AET3Gv20620600*	YSK2	364,603,011–364,610,005(−)	648	67.75	215	22.24	7.19	Cytoplasm Nucleus
*AetDHN2* ^*a*^	*AET3Gv20881700*	SK2	513,201,759–513,203,320(−)	396	66.41	131	13.27	10.87	Cytoplasm
*AetDHN3*	*AET4Gv20132600*	Y2SK3	41,638,766–41,640,754(−)	1383	67.97	460	44.58	9.03	Cytoplasm
*AetDHN4–1*	*AET5Gv20866700*	YSK2	458,683,120–458,684,124(+)	432	71.53	143	14.52	8	Cytoplasm
*AetDHN4–2*	*AET5Gv20866800*	YSK2	458,689,123–458,689,989(+)	402	69.65	133	13.93	9.44	Cytoplasm
*AetDHN5*	*AET5Gv20990000*	YSK2	498,787,518–499,029,693(−)	465	67.1	154	15.56	10.16	Cytoplasm
*AetDHN6* ^*a*^	*AET6Gv20153700*	K1	34,801,689–34,803,209(−)	270	66.3	89	8.86	9.64	Cytoplasm
*AetDHN7*	*AET6Gv20653900*	SK3	352,066,646–352,068,329(−)	789	63.88	262	28.16	4.97	Nucleus
*AetDHN8*	*AET6Gv20864100*	K6	458,864,577–458,866,768(+)	1176	66.75	391	38.89	7.59	Cell wall Cytoplasm Nucleus
*AetDHN9*	*AET6Gv20864400*	K2	459,070,823–459,071,706(+)	282	63.12	93	9.66	7.43	Cytoplasm
*AetDHN10* ^*a*^	*AET6Gv20864500*	K9	459,131,190–459,134,316(+)	2190	63.56	729	75.05	6.35	Cytoplasm
*AetDHN11* ^*b*^	*AET6Gv20864900*	YSK1	459,422,062–459,423,545(+)	444	67.79	148	16.32	10.99	Cytoplasm Nucleus
*AetDHN12–1*	*AET6Gv20865700*	YSK2	459,787,474–459,841,184(+)	468	65.6	155	15.73	9.41	Cytoplasm
*AetDHN12–2*	*AET6Gv20866000*	YSK2	459,889,128–459,890,166(−)	483	66.87	160	16.26	8.94	Cytoplasm
*AetDHN12–3*	*AET6Gv20866400*	YSK2	460,020,381–460,021,566(−)	504	66.27	167	16.71	8.05	Cytoplasm
*AetDHN13*	*AET7Gv21347200*	K2	641,274,370–641,275,124(+)	339	63.13	112	11.53	6.8	Cytoplasm

*Ta T.aestivum*, *Td T.dicoccodies*, *Tu T.urartu*, *Aet Ae. tauschii*, *DHN* Dehydrin, “^a^”: truncated genes, “^b^”: potenial misannotated genes; *BP* coding sequence length, *AA* amino sequence length, *MW* molecular weight, *pI* isoelectric point

**Fig. 1:**
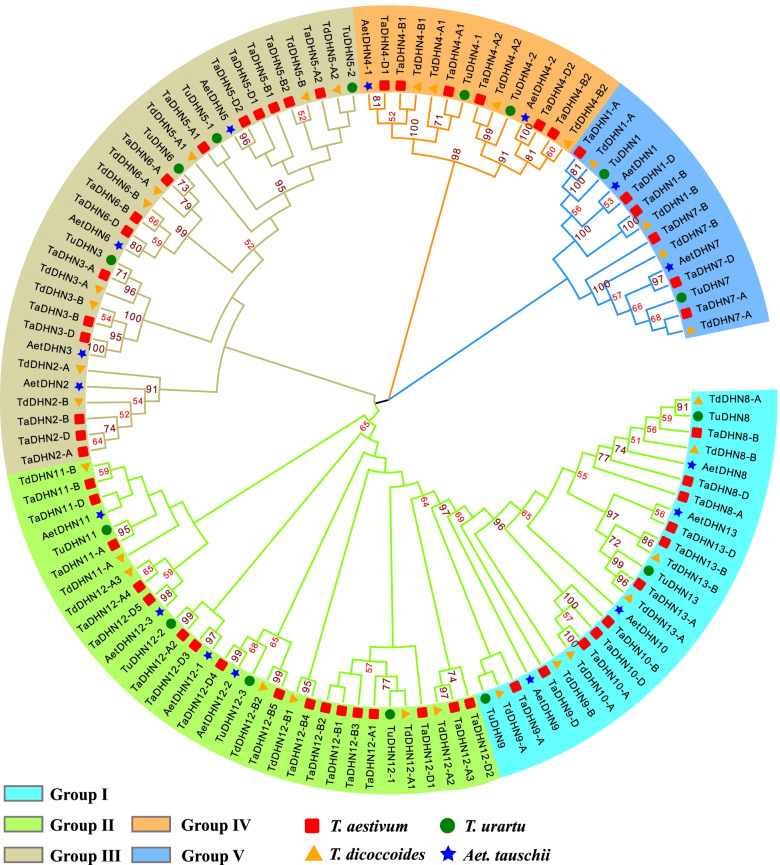
Phylogenetic analysis of the DHN genes in *Triticum aestivum* and its relatives. The phylogenetic tree was constructed using the neighbor-joining method and MEGA-X software; bootstrap scores > 50% are displayed; Different symbols represent different species, and different background colors represent different groups

To study the phylogenetic relationships of the DHN family, we constructed an unrooted phylogenetic tree using the 117 DHN protein sequences of bread wheat and its relatives (Fig. 1). The *DHN* genes were clustered into five major groups. Group I contained *DHN8/9/10/13*, which encode Kn type DHNs (Table 1). The K-segment copies varied from 2 to 14. Group II contained *DHN11/12*, which encode YnSKn type DHNs (except *TaDHN12-A3* encodes a SK2 type DHN), mainly the YSK2 type (Table 1). Group III contained *DHN2/3/5/6*, which encode YnSKn type DHNs (except *TdDHN3-A*, *TdDHN5-A1*, *AetDHN2*, and *AetDHN6*). Group IV contained the *DHN4* genes, which encode YSK2 type DHNs. The remaining clade was Group V, containing *DHN1* and *DHN7*, which encode YSK2 and SK3 type DHNs, respectively (Fig. 1 and Table 1).

We analyzed all DHN protein sequences in wheat and its relatives and identified three types of DHNs (YnSKn, SKn, and Kn). YnSKn was the most common with 81 among 117 DHNs, followed by Kn with 26, and only 10 *DHN* genes encoded SKn type proteins (Table 1). We also studied the phylogenetic relationships of *DHN* genes in wheat and its relatives, rice, and *A. thaliana*. Rice and *A. thaliana* had far fewer *DHN* genes than wheat and its relatives, with most being clustered into Group V, while several others belonged to Group I, Group III, and Group IV (Fig. S1). Therefore, the results showed that the *DHN* gene family conservation is limited to the close relatives of wheat, and very different from non- Triticeae species.

### Chromosomal distribution and synteny analysis of the DHN genes

**Fig. 2:**
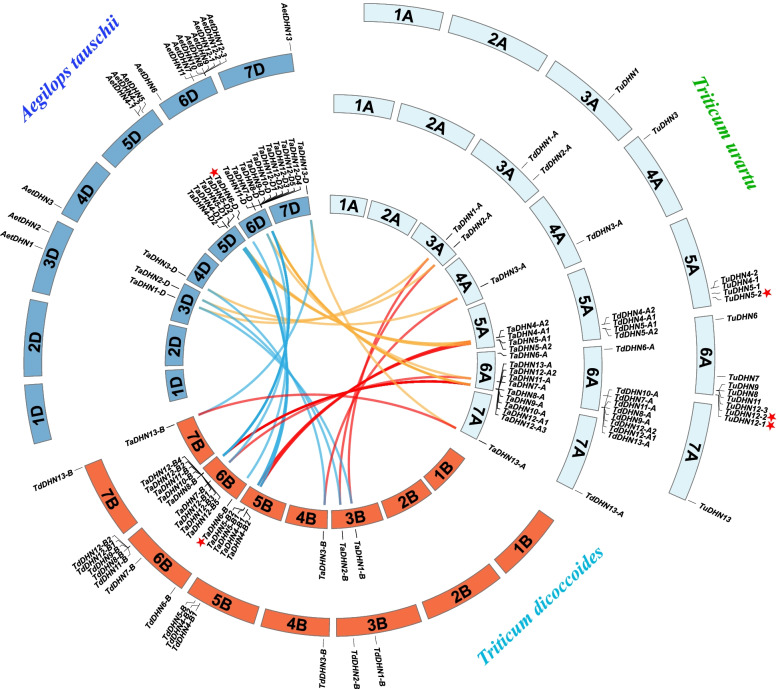
Synteny analysis and chromosomal distribution of DHN genes in bread wheat and its relatives. The hexploid bread wheat (*Ta*) and tetraploid durum wheat (*Td*) genomes were split into three and two diploid subgenomes, respectively. The A, B, and D genomes are represented by different colors. The DHN genes were assigned to the corresponding chromosome, according to their genome annotation file, except *TaDHN6B/D*, *TuDHN5–2*, and *TuDHN12–1/2*, which are labeled with a red star, and their positions are predicted by their homologs. Syntenic DHN gene pairs belonging to the same linkage groups between A and B, A and D, and B and D are linked with orange, red, and blue lines, respectively

To analyze the *DHN* gene syntenic relationships between bread wheat and its relatives, we identified orthologous genes among these four released species genomes. There were 18 *TaDHN*s, 17 *TdDHN*s, and 15 *TuDHN*s in the A sub-genome; 18 *TaDHN*s and 14 *TdDHN*s in the B sub-genome; and 19 *TaDHN*s and 16 *AetDHN*s in the D sub-genome. We identified and mapped the gene pairs of *Ta/Td/Tu-A*, *Ta/Td-B*, and *Ta/Aet-D* to corresponding genomic chromosomes (Fig. 2 and Table S1). Five genes, *TaDHN6B/D*, *TuDHN5–2*, and *TuDHN12–1/2*, were not assigned to chromosomes in the genome annotation file we used. We re-assigned these genes to the corresponding chromosomes based on the homologous and phylogenetic relationships (Table S1 and Fig. 1) and genomic location information (Table 1) of all *DHN* genes between the different diploid sub-genomes (Fig. 2). The *DHN* genes were distributed in the third to seventh homologous groups of bread wheat and its relatives, of which the fourth and seventh homologous groups had only one gene copy (except *T. urartu*-3A, with a missing gene), and the sixth homologous group had the most DHN genes, ranging from 7 to 14.

We also performed gene specific SSRs mining analysis for *TaDHN* genes, and 31 gene specific SSRs were discovered. These SSRs were distributed in the following four classes: di (dinucleotide), tri (trinucleotide), tetra (tetranucleotide) and penta (pentanucleotide) (FigureS2 and Table S2). Di SSR repeats (~ 67.74%) were far more than other repeats, and the tri SSRs (~ 25.81%) were found to be more than tetra (~ 3.22%) and penta repeats (~ 3.22%) (Fig. S2). After due validation, the predicted genes specific SSRs can be utilized for marker-assisted breeding programs in the future.

### Sequence analysis and re-annotation of the DHN gene family

**Fig. 3:**
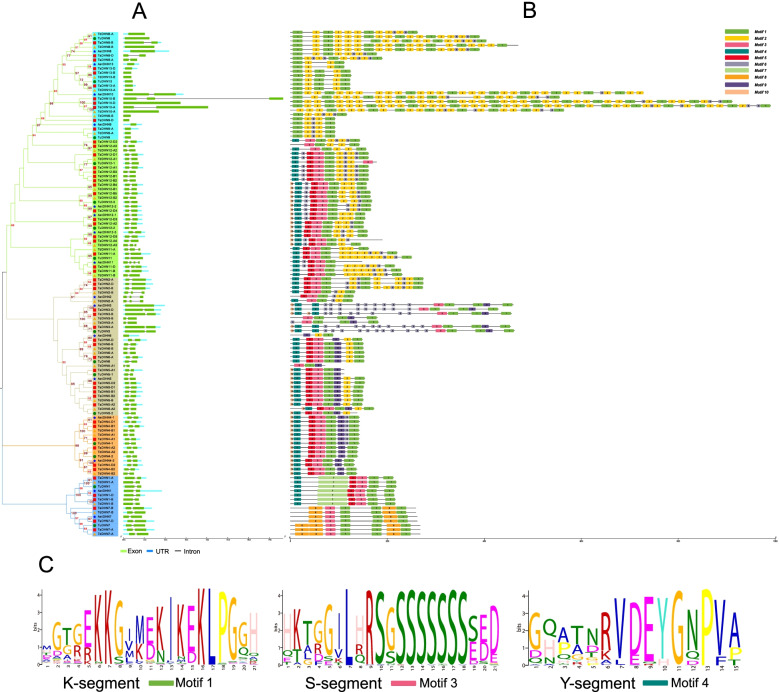
Sequence information of the DHN genes among bread wheat and its relatives. **A** Exon-intron structures. Green boxes, light blue boxes, and black horizontal lines indicate exons, UTRs, and introns, respectively. **B** Conserved domain composition. Dehydrin core domain (K-segment) and the other two dehydrin-related domains (S-segment and Y-segment) are shown in different colors. **C** Logos of the dehydrin-conserved domains

We collected the structural information of all *DHN* genes in the annotation file and visualized it using the Gene Structure Display Server (GSDS) web tool. The structural analysis results showed that the exon number varied between one and four. After analyzing the conserved domains of all DHN proteins, we found that all contained one dehydrin core motif (K-segment), but different numbers of Y−/S-segments (Fig. 3B and C). The remaining motifs are shown in Fig. S3. We manually checked the coding sequences and amino sequences of all *DHN* genes among bread wheat and its relatives, and combined the results with those of the phylogenetic (Fig. 1), synteny (Fig. 2), and sequence structural (Fig. 3) analyses. The truncated genes and potential mis-annotated genes are identified and marked in Table 1, and the identified missing genes are shown in Table S1.

According to the ploidy of bread wheat and its relatives, we speculate that the theoretical numbers of *DHN* genes should be 17 in *Ae. tauschii*, 17 in *T. urartu*, 34 in *T. dicoccoides*, and 52 in *T. aestivum*. However, *TaDHN9-B*, *TdDHN5-B2/10-B/12-B3*, *TuDHN2/10*, and *AetDHN5–2* were missing genes, and with tandem duplication events occurring in *TaDHN12-A/B/D*, it resulted in the actual gene number deviating slightly from the theoretical gene number (Table S1). In the phylogenetic analysis (Fig. 1), genes belonging to the same group occasionally had individual genes encoding a DHN type that varied from most genes in the group (e.g., Group II genes mostly encoded YSK2 type DHNs, but *TaDHN12-A3* encoded SK2 type DHNs). After manually checking the sequence, we found that this occurred due to sequence truncation or potential mis-annotation. The first 28 amino acids of *TaDHN12-A3* were mis-annotated, resulting in a loss of the Y-segment.

### Analysis of cis-acting elements in the promoter regions of TaDHN genes

*DHN* genes play important roles in response to various stressors. *Cis*-acting elements control their target gene expression by interacting with transcription factors [39, 40]. Hence, identifying the *cis*-acting elements will help understand the potential regulatory mechanism. We analyzed the 1500-bp upstream region from the start codon (ATG) for putative *cis*-acting elements of all stress-responsive *TaDHN* genes, and identified eight different types of *cis*-acting elements (Fig. S4), Among them, the abscisic acid (ABA) responsive element (ABRE), the methyl jasmonate (MeJA) responsive element (MeJA-RE), and the TCA-element are involved in hormone signaling, whereas the drought responsive element (DRE1/DRE core), low temperature responsive (LTR), TC-rich, and MYB binding site (MBS) are involved in the abiotic stress response. The results show varied distribution and abundance of the *cis*-acting elements among the 55 *DHN* promoters (Fig. S4). The ABRE elements involved in ABA signaling and osmotic stress [41–43] appeared in all *DHN* gene promoter regions, and the DRE1/DRE core being abundantly present in 45 *DHN* promoter regions ensured that *DHN* gene expression was regulated in response to drought stress [44]. MeJA-RE appeared in the promoter regions of 41 *DHN* genes, followed by TC-rich repeats and LTR being present in 21. These three types of *cis*-acting elements also play critical roles in response to abiotic or biotic stress [45]. The MBS *cis*-acting element appearing in 16 *DHN* genes is important for the stress (esp. drought) response and ABA signaling [28]. Taken together, the wide distribution of various hormone and stress responsive elements in the promoter regions demonstrates that *DHN* genes are potentially involved in the environmental stress response in plants.

### Expression profile of DHN genes in different tissues and in response to various biotic stressors

**Fig. 4:**
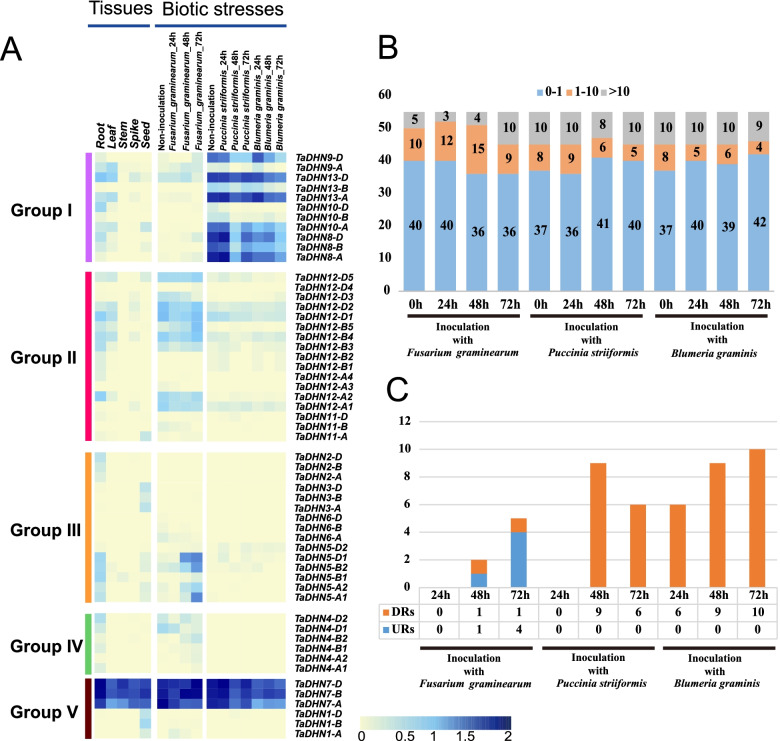
Expression analysis of *TaDHN* genes in various tissues and stress treatments. **A** The expression profiles of the *TaDHN* genes in different tissues under biotic stress. The expression data of 55 *TaDHN* genes were involved in roots, leaves, stems, spikes, seeds, and pathogen infection (*Fusarium graminearum*, *Fusarium* head blight (FHB); *Blumeria graminis*, powdery mildew; *Puccinia striiformis*, stripe rust) under different treatment times. **B** The number of *TaDHN* genes corresponding to the three expression levels (tpm: 0–1, 1–10, and > 10) under different pathogen infection times. The three expression levels are represented by different colors. (C) The number of up/down-regulated (URs or DRs) genes under different pathogen infection times. The DRs and URs are represented by orange and blue, respectively

We analyzed the RNA-seq data of five tissues/organs (roots, leaves, stems, spikes, and seeds) to characterize the expression of the bread wheat *DHN* genes. Of the 55 *TaDHN* genes, while 65% (*n* = 36) were expressed in at least one tissue, with a wide expression level range (tpm_max_ = 1–204) (Fig. 4A and Table S3), the remaining 35% showed no or very low expression (tpm_max_ < 1), like *DHN6* and *DHN13* (Fig. 4A and Table S3). About 48% (*n* = 26) of the *DHN* genes were expressed in roots (*DHN2/4/5* and some *DHN8/10/12* genes were expressed specifically in roots). Twelve *DHN* genes were expressed in seeds and leaves (*DHN1/3* genes were expressed specifically in seeds). Few *DHN* genes were expressed in stems (n = 3) or spikes (*n* = 4) (Fig. 4A and Table S3).

We also analyzed the RNA-seq data of bread wheat inoculated with *F. graminearum*, *B. graminis*, or *P. striiformis* to investigate how *TaDHN* genes function in response to biotic stress. About 55% (*n* = 30) of the *DHN* genes (*DHN4/5/7/8/9/10/12/13)* were expressed with a wide expression level range (tpm_max_ = 1–671) (Fig. 4A and Table S3). To accurately understand how *DHN* genes respond to different biotic stressors, we divided them into three categories according to gene expression levels (tpm): 0–1 (no to low), 1–10 (medium), and > 10 (high). Most of the *DHN* genes showed no or low expression, while many showed medium expression (Fig. 4B). Although the number of highly expressed genes changed with increasing inoculation time, the *DHN* gene family did not respond strongly when inoculated with *F. graminearum*, *B. graminis*, or *P. striiformis* (Fig. 4B).

We defined *DHN* genes with tpm fold change > 1 (treatment vs. control) and tpm value change > 10 as up- and down-regulated genes (URs and DRs) to further understand the *DHN* family gene expression changes under different biotic stressors. No URs or DRs were detected after 24 h in bread wheat inoculated with *F. graminearum*. There was one DR and one UR at 48 h and one DR and four URs at 72 h (Fig. 4C). *TaDHN5-D1* was up-regulated at 48 and 72 h, while *TaDHN5-A1/B2* and *TaDHN12-B5* were up-regulated only at 72 h (Fig. 4A and Table S3). However, no URs occurred in bread wheat inoculated with *B. graminis* or *P. striiformis*, and most genes were either down-regulated or had a very low tpm value (Fig. 4C).

### Expression profile of DHN genes in response to various abiotic stressors

**Fig. 5:**
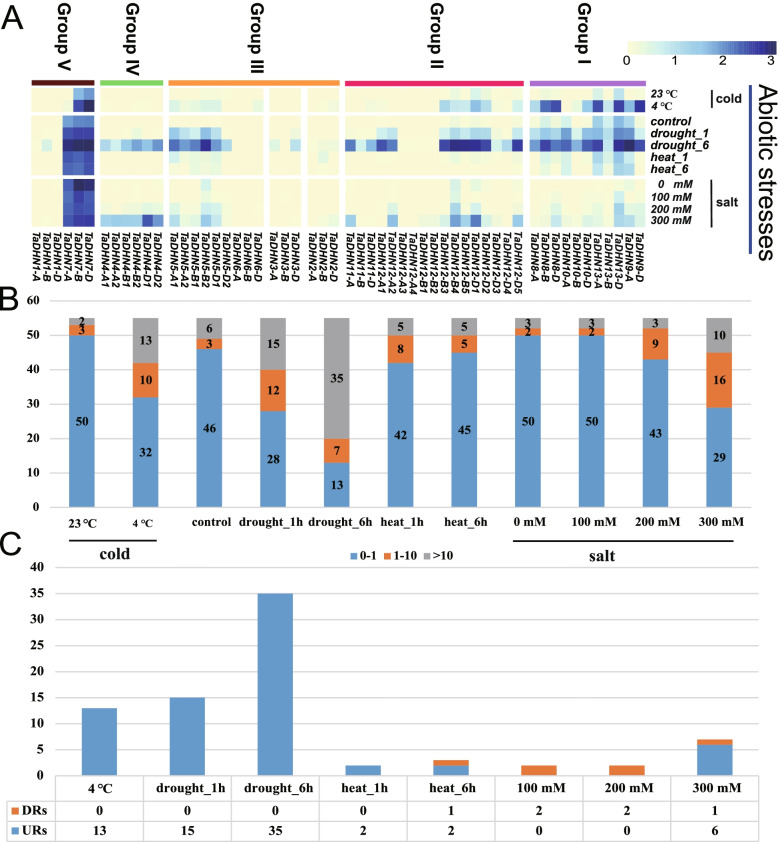
Expression analysis of the *TaDHN* genes under various abiotic stressors. **A** The expression profiles of the *TaDHN* genes under the abiotic stress treatments. The expression levels of 55 *TaDHN* genes changed in response to cold, drought, heat, and salt under different treatment times and concentrations. **B** The numbers of *TaDHN* genes corresponding to the three expression levels (tpm: 0–1, 1–10, and > 10) under different abiotic treatment times. The three expression levels are represented by different colors. **C** The number of up/down-regulated (URs or DRs) genes under different abiotic treatment times. The DRs and URs are represented by orange and blue, respectively

We analyzed the bread wheat RNA-seq data under cold, drought, heat, and salt conditions to understand how *TaDHN* genes respond to abiotic stress. Of the 55 *TaDHN* genes, 78% (*n* = 43) were expressed, and genes from *DHN1/2/3* showed no or very low expression (Fig. 5A and Table S3). We also analyzed the *DHN* family gene expression levels under the four different abiotic stressors. Ten *DHN* genes showed medium level expression under cold stress (1 < tpm < 10), while fifteen were highly expressed (tpm > 10) (Fig. 5B). Under drought stress, while twelve and fifteen genes had medium and high expression at 1 h, seven had medium and thirty-five had high expression at 6 h. When we subjected bread wheat to heat stress, only five *DHN* genes were highly expressed at 1 and 6 h, and eight and five were expressed at medium levels at 1 and 6 h, respectively (Fig. 5B). The *DHN* genes were mostly insensitive to 100 or 200 mM NaCl, as only three genes each was highly expressed at both concentrations, while only two and nine had medium expression levels at 100 and 200 mM NaCl, respectively. However, 10 and 16 *DHN* genes had high and medium expression levels, respectively, at 300 mM NaCl (Fig. 5B).

Then, we analyzed the DRs and URs of the *DHN* family in response to the four abiotic stressors. No DRs appeared in response to the cold or drought stressors, but we found 13 URs under cold stress. Most of the 15 and 35 URs responding to drought stress at 1 and 6 h, respectively (Fig. 5C), were highly expressed (Fig. 5A and Table S3). In summary, most *DHN* genes were insensitive to both heat and 100/200 mM NaCl stressors, as we detected very few DRs or URs under both these conditions. We also detected six URs under the 300 mM NaCl stress, indicating that *DHN* genes are sensitive to high salinity. Most of the Group I genes expressing Kn type proteins mainly under cold and drought stress were URs (Fig. 5A and C). In contrast, although some Group I *DHN* genes had high tpm values under the *B. graminis* and *P. striiformis* inoculation, surprisingly most were DRs (Fig. 4C).

### Response of DHN genes under various hormone treatments

**Fig. 6:**
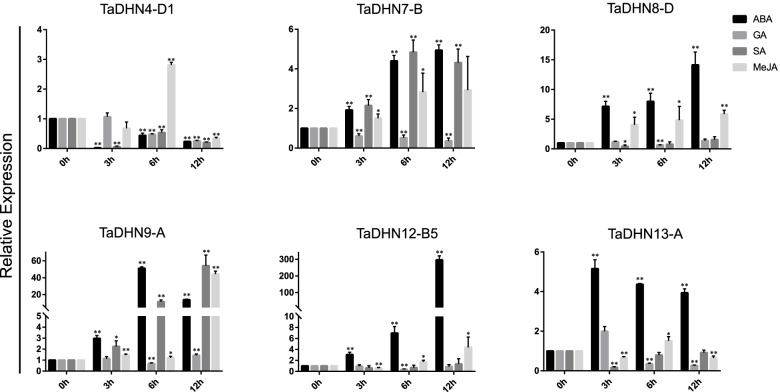
Expression analysis of six selected *TaDHN* genes under different hormone treatments. Expression profiles of six selected *TaDHN* genes (including *TaDHN4-D1*, *TaDHN7-B*, *TaDHN8-D*, *TaDHN9-A*, *TaDHN12-B5*, and *TaDHN13-A*) were analyzed under ABA (100 μM), GA (50 μM), SA (100 μM), and MeJA (100 μM) treatments. Error bars represent standard deviations (SDs) calculated from three independent biological replicates. *P* < 0.05 (*) and *P* < 0.01(**) by Student’s *t*-test

To understand the roles of the TaDHN genes in response to hormones, six TaDHN genes with higher expression levels under various stress conditions were selected to analyze their expression profiles. We found that all responded strongly to the ABA treatment (Fig. 6). Among them, while ABA treatment only down-regulated TaDHN4-D1 significantly (*p* < 0.01), it up-regulated the others. In contrast, gibberellin (GA) treatment weakly induced or inhibited the expression of DHN genes. While salicylic acid (SA) treatment significantly upregulated TaDHN7-B and TaDHN9-A (*p* < 0.01), it either down-regulated or did not affect the other genes (Fig. 6). However, MeJA treatment significantly induced all selected genes (*p* < 0.01 or *p* < 0.05); TaDHN4-D1 and TaDHN13-A peaked at 6 h, TaDHN9-A and TaDHN12-B5 peaked at 12 h, and TaDHN7-B and TaDHN8-D were up-regulated throughout the entire MeJA treatment period (Fig. 6). In summary, the DHN genes showed various expression patterns under different hormone treatments. All selected DHN genes were highly sensitive to the ABA treatment (particularly TaDHN9-A and TaDHN12-B5 with strikingly high expression). Since ABA signaling is very important in regulating plant stress response [41], the abundance of ABA-related cis-acting elements (Fig. S4) and the strong response of the DHN genes to the ABA treatment reflects the crucial role of the DHN gene family in various stress conditions.

### Interaction network and subcellular localization

In order to further understand how the abiotic stress-induced DHN proteins function, we used the STRING database to annotate the proteins encoded by the wheat *DHN* genes and their *Ae. tauschii* homologs (Table S4). Then, using the well-studied *AetDHN*s we constructed an interaction network (Fig. S5). We found that these DHN proteins were not only closely connected (except EMT32858 and EMT15121, which are annotated as cold shock proteins), but also their functions covered many aspects of wheat abiotic stress response. For example, *DHN8/9/10/14* encode cold shock proteins, *DHN4/12/13* encode salt-induced proteins (Fig. S5 and Table S4), while *DHN5/4–1/12* encode EMT-25371/30993/24840 that interact with a heat shock protein, EMT106830 (Fig. S5 and Table S4). Moreover, we speculate that DHN proteins can cooperate with each other when plants are under abiotic stress.

**Fig. 7:**
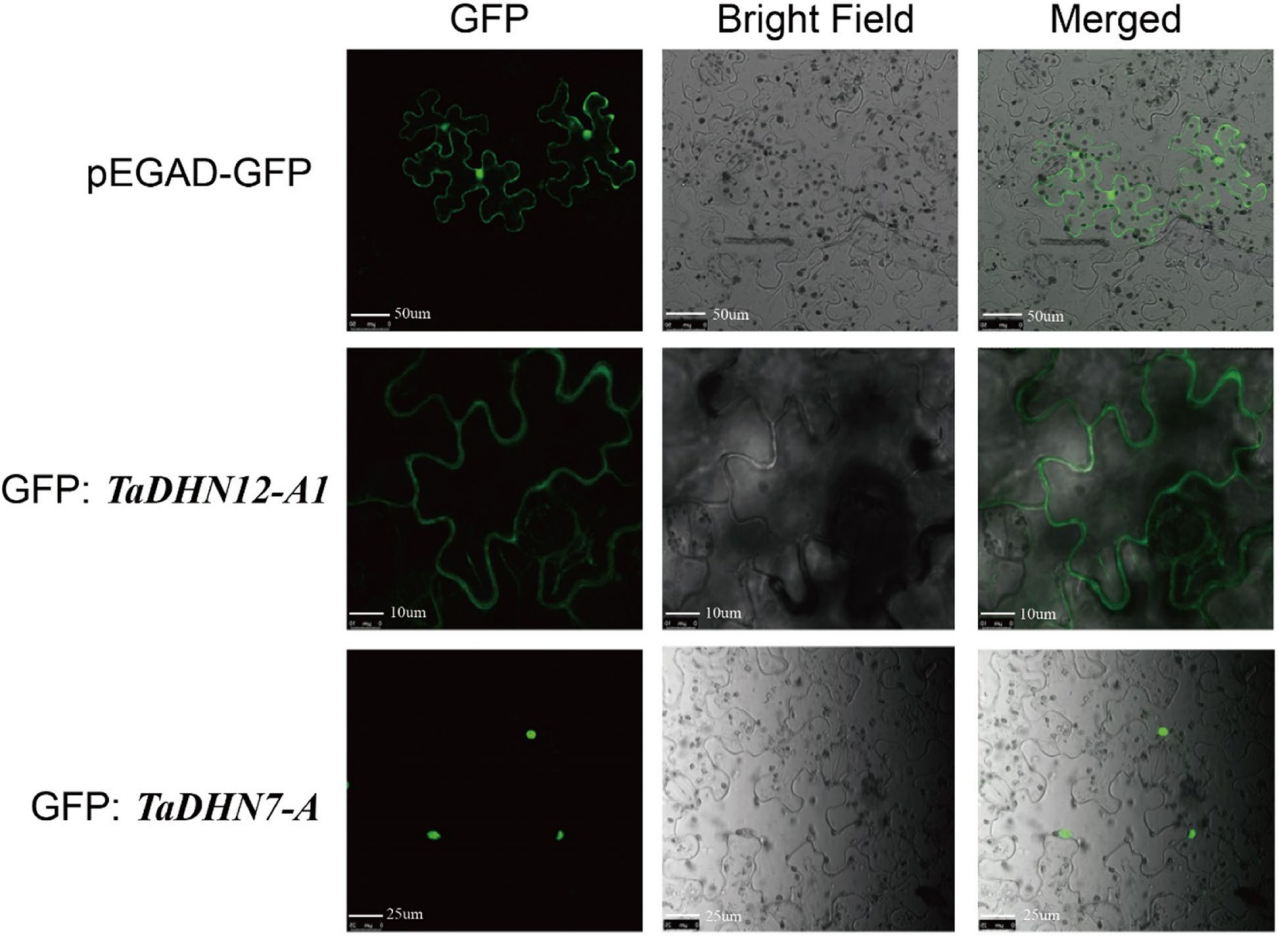
Subcellular localization of selected *TaDHN*-GFP fusion proteins in *N. benthamiana* leaf epidermal cells. *TaDHN12-A1* and *TaDHN7-A* were localized to the cytoplasm and nucleus, respectively. pEGAD-GFP was used as a positive control

Using bioinformatics, we also predicted the subcellular location of the DHN protein to understand where it might function (Table 1). In order to determine and experimentally verify the accuracy of the prediction, we selected two genes, *TaDHN12-A1* and *TaDHN7-A1*, and found that their encoded proteins were indeed located in the cytoplasm and nucleus, respectively (Fig. 7). The results thus verify and confirm the accuracy of the bioinformatics prediction.

## Discussion

Since many *DHN* genes are key to protecting plants from various environmental stressors, they are potential candidates for crop breeding and improvement. Using a comprehensive approach in this study, we identified 55 *DHN* genes in hexaploid bread wheat (*T. aestivum*), 31 in tetraploid durum wheat (*T. dicoccoides*), 15 in diploid *T. urartu*, and 16 in diploid *Ae. tauschii*.

### Identification of DHN genes in bread wheat and its relatives

According to the chromosomal distribution and homologous relationship of the *DHN* gene family (Fig. 2 and Table S1), they were unevenly distributed among different homologous groups, with most being distributed in homologous groups 5 and 6. We also found similar distribution in seven other sub-genomes (*Ta-A/B/D*, *Td−/A/B*, *Tu-A*, and *Aet-D*), thus providing high confidence for the identification. We also observed the translocation events occurred on *T. urartu* 4A chromosome; *TaDHN3-A/B/D*, *TdDHN3-A/B*, and *AetDHN3-D* were distributed on the homologous group 4 distal long arm; while *TuDHN3* was located on the distal short arm. A previous *T. urartu* genome study had already reported the translocation event [12]. The close relationships between homologous groups greatly improved the accuracy of identification. For example, based on the annotation file, *TuDHN5–2* was initially placed on the unmapped chromosome. But based on its high similarity with *DHN5* genes located on seven other sub-genomes, *TuDHN5–2* was re-assigned on the 5A chromosome. The missing genes were also identified according to the relationships between homologous groups, like *TuDHN2* was identified as a missing gene because although *TaDHN2-A/B/D*, *TdDHN2-A/B*, and *AetDHN2-D* were localized to homologous group 3, their corresponding homoeologs on chromosome 3A of *T. urartu* genome were absent. Similar gene loss is widespread in other gene families and may occur during the wheat polyploidization process [5, 46, 47]. Although genes were missing from some genomic regions, homologous relationships were clear among the *DHN* genes located in different diploid sub-genomes, indicating that this gene family is evolutionarily well-conserved. The gene families in different sub-genomes of polyploid plants, such as bread wheat, particularly small or medium-sized families, are conserved in number or sequences. Using the sequence similarity of the gene family between sub-genomes, we can accurately identify the target genes. Furthermore, since automated annotation generates truncated and mis-annotated genes, additional manual checking is necessary for proper identification.

### Evolution and expansion of the DHN genes among bread wheat and its relatives

Gene duplication occurs in different ways, including whole-genome duplication, segmental duplication, and single-gene duplication (including tandem and dispersed duplications) events. Duplication events are important in expanding a gene family [48–50]. According to the chromosomal distribution and syntenic relationships between bread wheat and its relatives, allopolyploid events were the main driving force behind expanding the hexaploid wheat *DHN* family. The *DHN4/5/12* genes have undergone tandem duplication events. Three *DHN12* genes occur in the diploid genome of wheat ancestors (one gene is missing in *Td-B*), while the *DHN12* gene number has changed in the three wheat sub-genomes (A, B, and D). The A sub-genome of bread wheat has one more copy than the A genome of the ancestors. The B and D sub-genomes have two more copies than the B and D genomes of the ancestors, respectively (Table S1). We analyzed the *TaDHN12* genes as tandems. Therefore, we speculate that the *DHN* gene family in bread wheat has undergone tandem duplication events after polyploidization, leading to more bread wheat *DHN* genes in the family than in its diploid donors.

The *DHN* family is a small family present in many plant species, like seven members in *Oryza sativa* [51], ten in *Arabidopsis* [52, 53], seven in *Pyrus pyrifoli* [54], and four each in *Vitis vinifera* and *V. yeshanensis* [16]. Bread wheat has the largest *DHN* gene number (55) among the above-mentioned plants. Even its diploid ancestors *T. urartu* and *Ae. tauschii* have greater number of *DHN* genes than other plant species, i.e., 15 and 17, respectively. Bread wheat has > 7.8 times higher number of *DHN* genes than rice, and this phenomenon cannot generally be explained by their ploidy. We hypothesize that the expanding *DHN* gene family may help Triticeae crops rapidly adapt to different stress conditions, particularly water-related stressors, like drought, therefore, contributing to the global distribution of bread wheat and its relatives. Whether we can detect *DHN* gene copy number variations in different wheat varieties is an interesting issue, with the recent release of the wheat pan-genomic data [55].

In the present study, tandem duplications occurred in linkage groups five and six, and tandem duplication genes (*DHN4/5/12*) appeared in clusters at corresponding chromosomes. The genes were combined with the expression profile results. Interestingly, while these tandem genes were mostly up-regulated under various abiotic stressors, the non-tandem genes like *DHN1/2/3/6* had no or very low expression (low tpm values), thus indicating that non-tandem genes are abiotic stress-insensitive. Notably, drought and cold stress up-regulated the *DHN 8/9/10/11* genes with high tpm values (Fig. 5A). These genes and the *DHN12* genes existed as gene clusters and were continuous in position (Fig. 2 and Table 1). These findings combined with the sequence characteristics indicate that these genes (*DHN8/9/10/11/12*) may have originated from tandem duplications of an ancestral gene. The need for ecological adaptability pushed them to subsequently evolve into the Kn and YSK2 groups. Adaptive evolution may have driven these tandem duplication events in the *DHN* gene family. Thus, tandem duplication events are the main reason for expansion of the *DHN* gene family in bread wheat diploid donors.

### Expression analysis of the TaDHN genes

We analyzed the expression profiles of the bread wheat *DHN* genes under various biotic and abiotic stress conditions. *TaDHN1/2/3/6* were insensitive to all the stress conditions in this study, with no or very low expression in all six tissues (Fig. 4A and Fig. 5A). The *TaDHN4* genes had root-specific expression and were mainly drought- and salt-inducible (Fig. 4A). Previous studies demonstrated that the *TaDHN5* genes and their homologs contributed towards drought and salt tolerance [56, 57]. This is consistent with our expression profile analysis results, which showed *TaDHN5* genes were indeed drought and salt stress-inducible (Fig. 5A). Some *TaDHN5* genes were also biotic stress-inducible (inoculation with *F. graminearum*), indicating that these genes may help in resistance against *F. graminearum*.

The *TaDHN7* genes were generally highly expressed (high tpm values) under all stress conditions (except *DHN7-A* in cold stress) and were constitutively expressed in all tissues (Fig. 4A and Fig. 5A). Among all *DHN* genes in bread wheat, *TaDHN7-A/B/D* are the only three genes that encode SK3-type proteins (Table 1). A previous study identified many SK3-type *DHN* genes having important functions under various abiotic stress conditions. For example, overexpression of *ShDHN* in tomato not only improves drought and cold stress tolerance, but also seedling growth under osmotic and salt stress [33]. Overexpression of *MusaDHN-1* in banana improves drought and salt stress tolerance [58]. A functional analysis demonstrated that *SpDHN1* in *Stipa purpurea* is important in drought stress resistance [59]. These studies of SK3-type DHN proteins indicate that *TaDHN7* genes may also be important in bread wheat facing various abiotic stressors. Taken together, *TaDHN* genes mainly responded to cold, drought, and high salinity stressors, but were insensitive to heat, low or medium salinity, and most biotic stressors.

## Conclusions

We comprehensively analyzed the *DHN* gene family, using molecular characterization, phylogenetic classification, chromosomal distributions, gene structure, conserved motifs, and missing, truncated, and mis-annotated genes, as well as *cis*-acting elements. Based on six RNA-seq datasets, the *DHN* genes exhibited distinct tissue-specific expression patterns, and we identified the induced genes under different stress conditions. Conserved, duplicated *DHN* genes may be important in helping wheat adapt to various conditions, therefore, contributing to its distribution as a global staple food. The cooperation of multiple DHNs may be important in protecting plants from abiotic stress. Therefore, our study results will not only help in further study of the stress resistance mechanisms of the *DHN* gene family, but also facilitate wheat breeding by fine-tuning its important traits.

## Methods

### Plant materials

The bread wheat variety “Chinese Spring” and N. benthamiana were used for RT-PCR and subcellular localization, respectively. And these materials are presented from State Key Laboratory of Crop Biology, College of Agronomy, Shandong Agricultural University (Taian, China).

### Sequence search, identification, and naming of the DHN genes

The genome sequences and gene annotations of bread wheat (*T. aestivum*) and wild emmer wheat (*T. dicoccoides*) were obtained from the Ensemble Plants website (http://plants.ensembl.org/) [60]. The genome files for *T. urartu* and *Ae. tauschii* were obtained from the (http://www.mbkbase.org/Tu/) and (http://aegilops.wheat.ucdavis.edu/ATGSP/annotation/) websites, respectively (Table S5) [13, 61]. To identify the DHN genes in bread wheat and its relatives, HMMER 3.1 (http://www.hmmer.org/) with default parameter settings and the BLAST algorithm for proteins (BLASTP) with the threshold expectation value set to 1E-20 were performed using the hidden Markov model (HMM) (version 3.0) profiles of the dehydrin domain (PF00257) obtained from the Pfam database (http://pfam.xfam.org/) as the query [62, 63]. We merged all hits obtained and removed the redundant hits. All non-redundant protein sequences were further analyzed with the NCBI conserved domain database (CDD, https://www.ncbi.nlm.nih.gov/cdd) and InterPro (http://www.ebi.ac.uk/interpro/) to confirm the conserved domain of the DHN protein in each candidate sequence [64, 65]. The methodology flowchart of the identification of DHN gene family was also provided (Fig. S6). Tandem genes were screened by a custom Perl script, according to the following standards: (i) length of alignable sequence covers > 70% of longer gene; (ii) similarity of aligned regions > 70%; (iii) The physical distance between the align genes on the chromosome < 500 kb.

We suggest a consistent naming pattern for all DHN genes of bread wheat and its relatives, considering the genomic location and phylogenetic and syntenic relationships of the DHN genes between different diploid sub-genomes (*Ta/Td/Tu-A*, *Ta/Td-B*, and *Ta/Aet-D*). (i) Each DHN gene name starts with an abbreviation for the species name. For example, *T. aestivum* (Ta), followed by the abbreviation of dehydrin gene family: DHN; (ii) the gene names include an A, B, or D, indicating the sub-genome where they are located. For example, *TaDHN1-A*; (iii) putative homologs between sub-genomes have identical gene names except for the sub-genome identifier or species name (e.g., *TaDHN7-A*, *TaDHN7-B*, *TaDHN7-D*, *TdHN7-A*, *TdDHN7-B*, *TuDHN7*, and *AetDHN7*); (iv) tandem genes are consecutively numbered (e.g., *TaDHN4-A1* and *TaDHN4-A2*).

### Phylogenetic and synteny analysis

All identified DHN protein sequences were aligned using the MUSCLE [66] program with default parameters. Phylogenetic trees were constructed using MEGA X software with the neighbor joining method and the following parameters: bootstrap (1000 replicates) and the Jones-Taylor-Thornton substitution model [67].

All identified DHN genes in wheat and its relatives were located on pseudo-chromosomes based on the physical location information acquired from the genomic database. To understand the relationship between the DHN genes identified in wheat and its relatives at the genomic level, the hexploid bread wheat (*Ta*) and tetraploid durum wheat (*Td*) genomes were split into three and two diploid sub-genomes (AA, BB, DD and AA, BB), respectively. A collinear analysis was performed using the five sub-genomes with diploid *T. urartu* and *Ae. tauschii* genomes and JCVI software (https://github.com/tanghaibao/jcvi/wiki). The results were visualized by Circos [68].

### Analysis of DHN gene characteristics and SSRs mining

Isoelectric points and molecular weights were determined using ExPASy (https://web.expasy.org/protparam/). Subcellular localization of all DHN genes was predicted using the Cell-PLoc (version 2.0) website (http://www.csbio.sjtu.edu.cn/bioinf/Cell-PLoc-2/) [69]. Exon-intron structures of the DHN genes in bread wheat and its relatives were displayed using the Gene Structure Display Server (GSDS, http://gsds.gao-lab.org/index.php) [70]. The promoter sequences (1500-bp upstream of the ATG translation start codon) of the DHN genes were extracted from the bread wheat genome sequence (IWGSC v1.0). *Cis*-acting elements were predicted in the PlantCARE database (http://bioinformatics.psb.ugent.be/webtools/plantcare/html/) [39], and the promoter sequences was provided (Table S6). The SSRs mining analysis was performed by GMATA software [71], and the specific markers were developed by Primer-BLAST [72].

### Expression profiles of the DHN genes in RNA-seq

To understand the expression profiles of the DHN genes in different tissues and under different stress conditions, six transcriptome datasets were downloaded from the NCBI (https://www.ncbi.nlm.nih.gov/) with accession numbers SRP043554, SRP045409, SRP300360, SRP041017, ERP013829, and ERP107574.

The RNA-seq data accession numbers SRP043554, SRP300360 and ERP013829 involved cold, salt and FHB infections. The SRP045409 data involved drought and heat stress. The SRP041017 data involved stripe rust and powdery mildew. The ERP107574 data were collected from various bread wheat tissues. The expression levels of the DHN genes were quantified as transcripts per kilobase million (TPM). The tpm value was calculated using Kallisto software [73].

### Plant cultivation, RNA isolation and RT-PCR

To investigate the expression patterns of the DHN genes in wheat under different hormone treatments, *T. aestivum* cv. Chinese Spring was used for the reverse transcription-polymerase chain reaction (RT-PCR) analysis. Bread wheat was planted in a growth chamber at 23 °C under a 16 h/8 h (light/dark) photoperiod. Then, 2-week-old seedlings were transferred to a hormone treatment solution containing 100 μM ABA, 50 μM GA, 100 μM SA, or 100 μM MeJA. The leaf tissues were harvested at 0, 3, 6, and 12 h and stored at − 80 °C after being frozen in liquid nitrogen. Total RNA of all samples was extracted using the RNAprep Pure Plant Kit (TIANGEN, Beijing, China) according to the manufacturer’s instructions. cDNA was generated with a one-step reverse transcription kit (TIANGEN). The Lightcycler 96 system (Roche, Mannheim, Germany) was used for the RT-PCR assay with the SYBR qPCR Master Mix (Vazyme, Nanjing, China); three technical replicates were carried out. Primer information could be found in supplementary (Table S7).

### Interaction network construction and subcellular localization

STRING website (https://string-db.org/) was used to analyze the interaction of DHN proteins with a confidence parameter set at 0.4 threshold [74]. Gene-specific primers were designed to amplify the coding sequences of the two selected *TaDHN* genes (**Table S****8**). Amplified fragments were ligated in-frame to the 5′-terminus with the expression vector pEGAD-GFP. Then, Constructed plasmids were infiltrated into abaxial air space of six-week-old *N. benthamiana* leaves using the transformed Agrobacterium strain *GV3101*. Infiltrated parts of the leaves were marked and fluorescence was observed under the confocal laser scanning microscope (Leica, German) after 48 h of infiltration.

## Supplementary Information

**Additional file 1.**

**Additional file 2.**

## Data Availability

The datasets used during the current study are available from NCBI (https://www.ncbi.nlm.nih.gov/) with accession numbers SRP043554, SRP045409, SRP300360, SRP041017, ERP013829, and ERP107574. The materials (“Chinese Spring” and *N. benthamiana*) used to support the findings of this study are available from the corresponding author upon request.
