# Supplementary material for: Genome-wide survey of the dehydrin genes in bread wheat (Triticum aestivum L.) and its relatives: identification, evolution and expression profiling under various abiotic stresses
Source: BMC Genomics. 2022 Jan 23;23:73. doi: 10.1186/s12864-022-08317-x (PMC8784006; doi:10.1186/s12864-022-08317-x)
Supplement: Supplementary file 1 — Additional file 1. [file 12864_2022_8317_MOESM1_ESM.docx]

**SUPPLEMENTARY INFORMATION**

**Genome-wide survey of the dehydrin genes in bread wheat (Triticum aestivum L.) and its relatives: identification, evolution and expression profiling under various abiotic stresses**

Yongchao Hao^1#^, Ming Hao^2#^, Yingjie Cui^1^, Lingrang Kong^1^, Hongwei Wang^1*^

^1^State Key Laboratory of Crop Biology, College of Agronomy, Shandong Agricultural University, Taian 271018, China

^2^College of Forestry, Shandong Agricultural University, Taian 271018, China

#These authors contributed equally to this work

^*^ Correspondence should be addressed to: Hongwei Wang (wanghongwei@sdau.edu.cn)


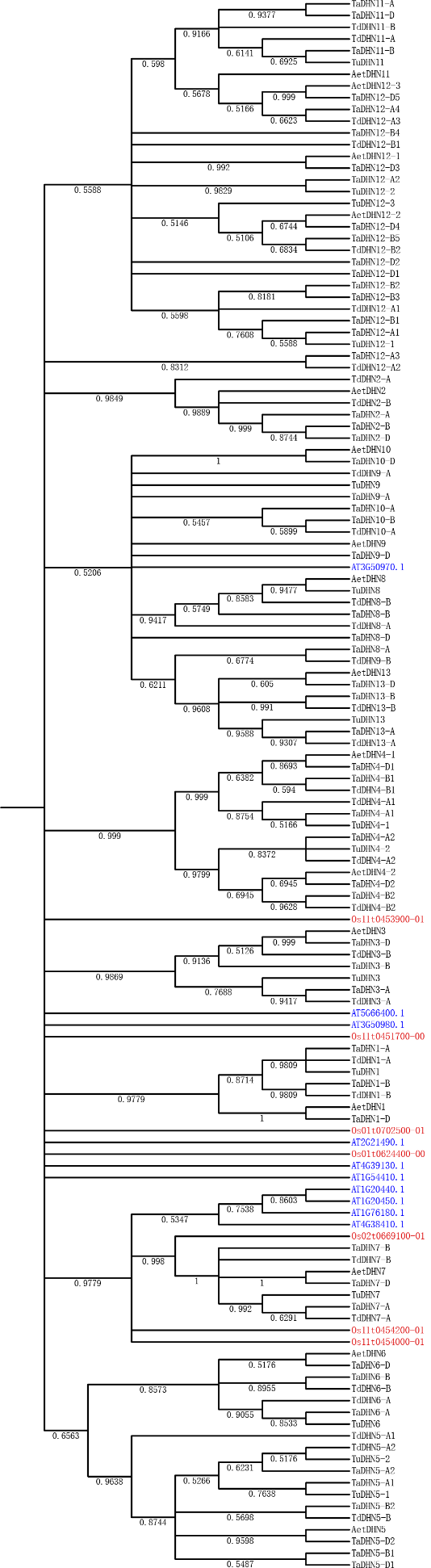


**Figure S1.** Phylogenetic analysis of the DHN genes between *Triticum aestivum* and its relatives, rice and *Arabidopsis thaliana*.


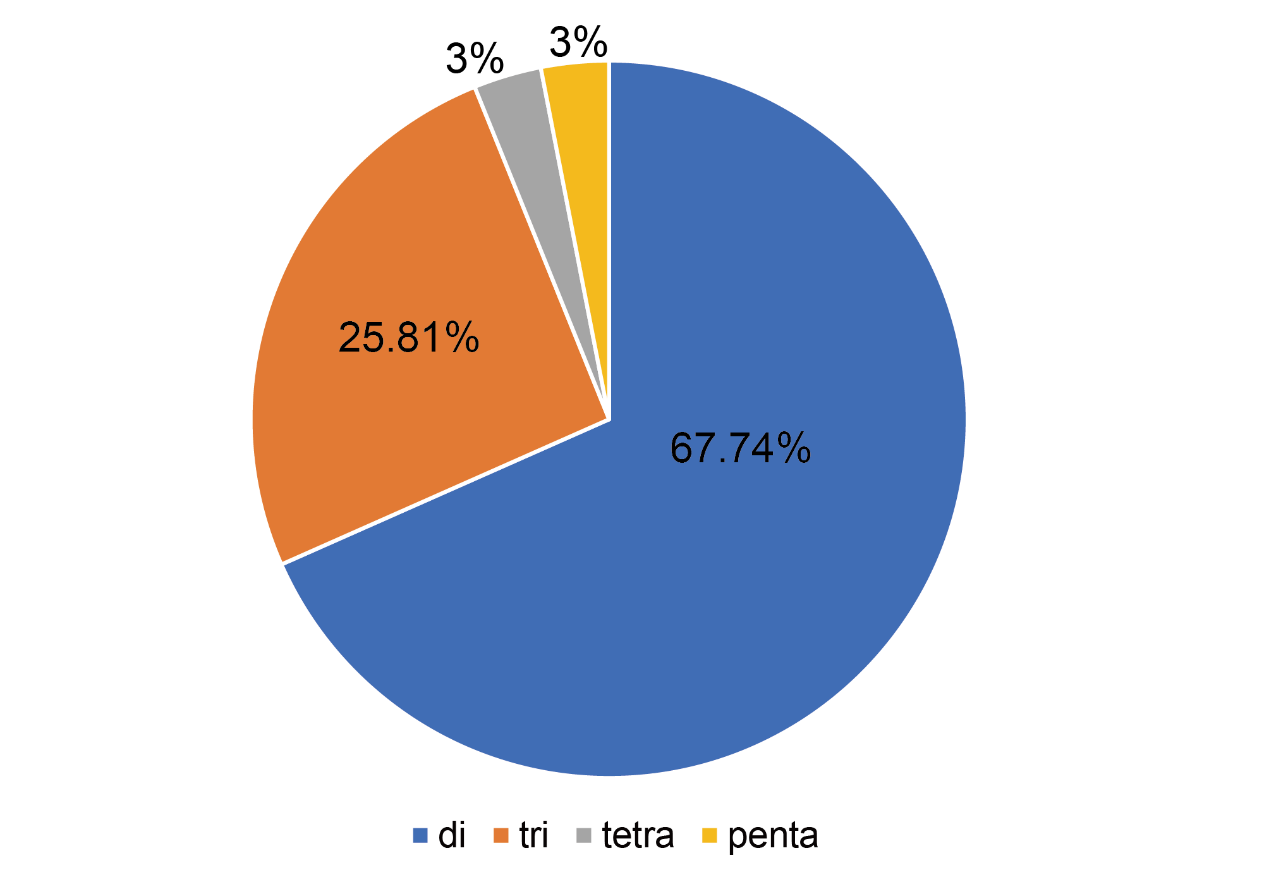


**Figure S2.** Proportion of four different gene specific SSRs. The di-, tri-, tetra- and penta- SSRs are represented by blue, orange, grey and yellow, respectively.


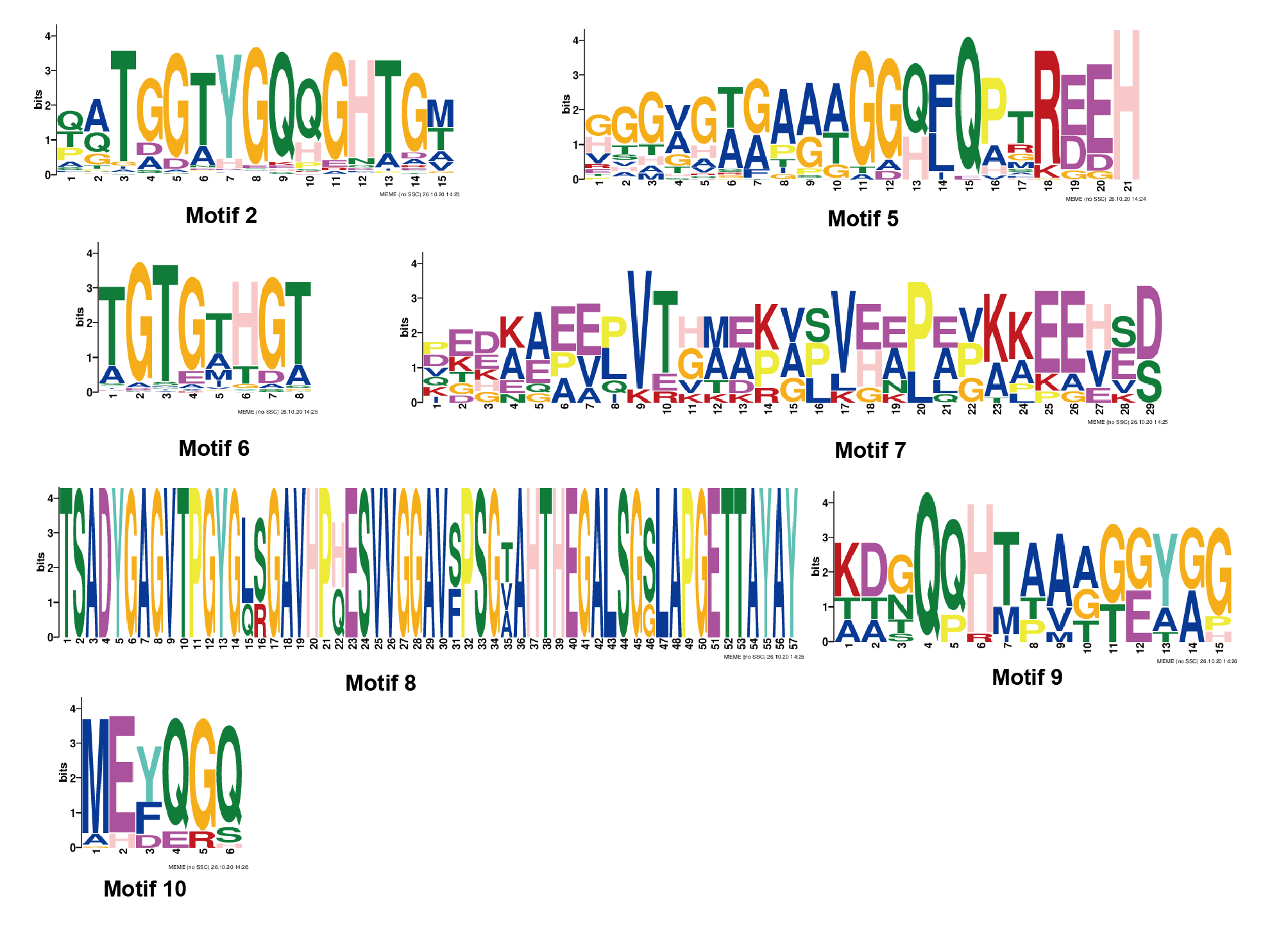


**Figure S3.** Logos of the conserved motifs. The Multiple EM for Motif Elicitation (MEME) program was used to determine the conserved protein motifs of DHN genes.


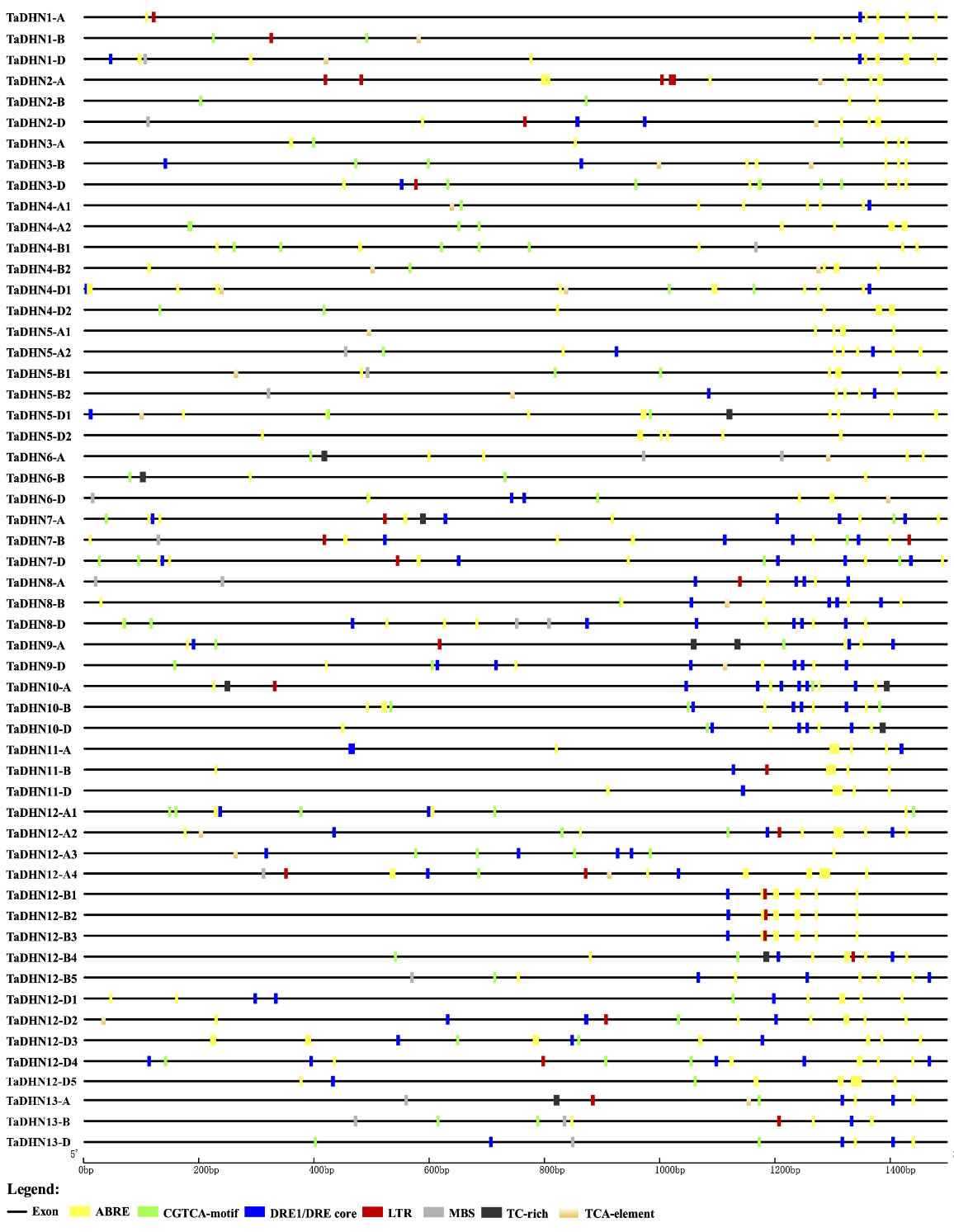


**Figure S4.** Potential cis-acting elements in the *TaDHN* gene promoter regions. Eight stress-related cis-acting elements in the *TaDHN* gene promoter regions were analyzed. Different cis-acting elements are represented by boxes with different colors.


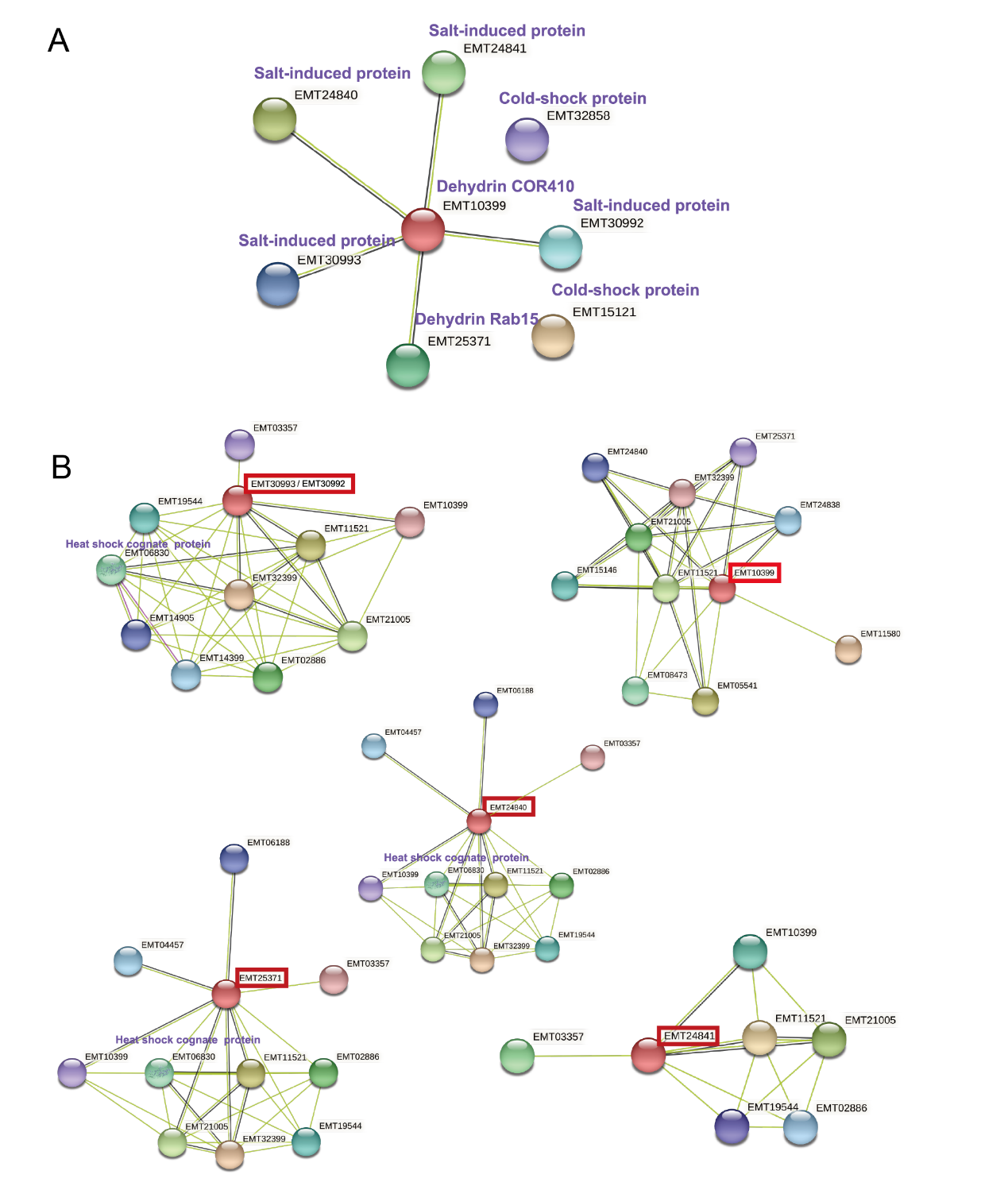


**Figure S5.** Interaction network of DHN proteins. (A) Interaction network of selected DHN proteins. The blue letters represent the annotation of these proteins. (B) The interaction networks of different DHN proteins. The selected DHN proteins were surrounded by red box, the blue letters represent the annotation of the protein which interacted with DHNs.


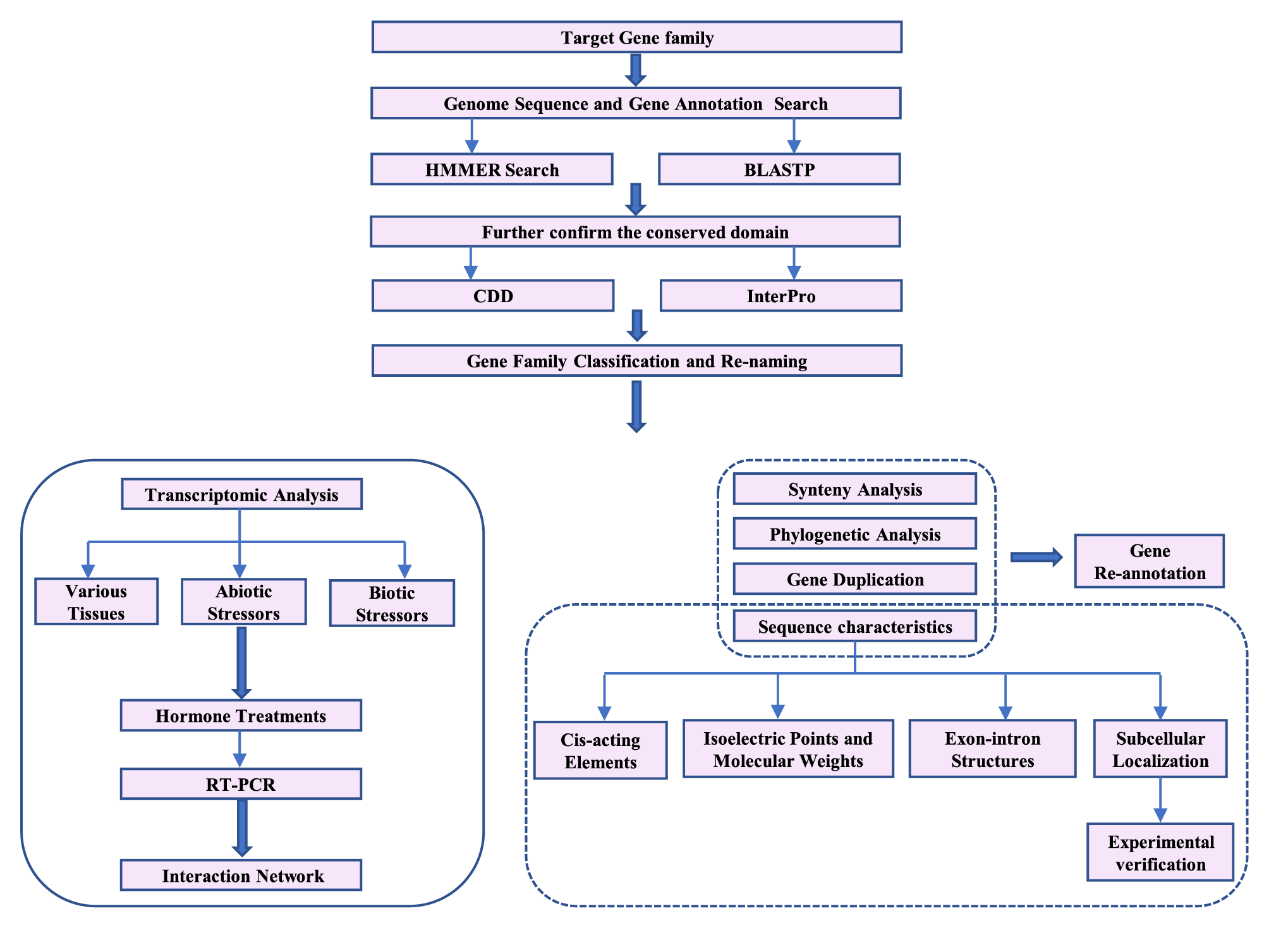


**Figure S6.** Flow chart of methodology pipeline in this study.
